# Eosinophilic Colitis as a Rare Etiology of Chronic Diarrhea: A Case Report

**DOI:** 10.7759/cureus.102607

**Published:** 2026-01-29

**Authors:** Hanane Aksim, Mohamed Amine Haouane, Meryem Belhamdiya, Mohamed Amine Azami, Khalid Gharbi, Rachid Akka

**Affiliations:** 1 Hepatogastroenterology, Avicenne Military Hospital, Marrakech, MAR; 2 Pathology, Cadi Ayyad University of Marrakech/Avicenne Military Hospital, Marrakech, MAR

**Keywords:** case report, chronic diarrhea, corticosteroids, eosinophilic colitis, eosinophilic polymorphonuclear cells

## Abstract

Eosinophilic colitis (EC) is a rare condition associated with abnormal infiltration of the colonic mucosa by eosinophilic polymorphonuclear cells (EPS). The clinical picture in adults with EC is varied and nonspecific, with the most common symptoms being diarrhea and abdominal pain. Although the etiology of primary colonic eosinophilia is unknown, several criteria are involved in the pathogenesis of secondary eosinophilic colonic infiltration (parasitic infections, drugs, and food allergens), which must be excluded to diagnose the primary form of the disease correctly. The diagnosis of EC currently poses a major problem due to the lack of consensus on histological diagnostic criteria for the disease and on the physiological levels of eosinophils (EPS) in normal colonic mucosa. Endoscopy, imaging, and laboratory tests may be useful in ruling out other similar conditions, but EC remains a diagnosis of exclusion. Several treatment options are available, but most of the evidence comes from case reports and small case series, which limits their value. We report here the case of EC in a 67-year-old female patient, which was revealed during an etiological evaluation of chronic diarrhea. Endoscopy revealed a normal macroscopic appearance of the mucosa of the stomach, duodenum, terminal ileum, and colon. The patient was treated with systemic corticosteroids and antihistamines, with good clinical progress, and the episodes of diarrhea decreased after one week. This case highlights the value of treatment with oral corticosteroids and antihistamines and also reminds us of the important role of endoscopy in the etiological diagnosis of chronic diarrhea.

## Introduction

Eosinophilic gastrointestinal disorders (EGIDs) are defined as disorders that affect the digestive tract and are characterized by eosinophil-rich inflammation in the absence of known causes of eosinophilia (e.g., parasitic infections, malignant causes, drug reactions, etc.). They include eosinophilic gastroenteritis (EGE), eosinophilic esophagitis (EOE), and eosinophilic colitis (EC), which is the rarest. Among EGIDs, EoE is the most frequently diagnosed, followed by EGE and EC, with estimated prevalences of 39-56.7, 6.4, and 3.5 cases per 100,000 population, respectively [1]. Although the incidence of EGIDs has increased since the 1990s, these conditions remain rare, and evidence-based management strategies are limited. Consequently, clinical decision-making is often guided by data derived from case reports and small case series. We report a case of EC identified during the etiological workup of chronic diarrhea, highlighting the diagnostic approach and response to corticosteroid therapy.

## Case presentation

We report the case of a 67-year-old woman admitted to our clinic for the etiological assessment of chronic diarrhea that had been ongoing for more than six months. The patient has a history of uveitis and is under treatment, with no known drug allergy or herbal allergy and no similar case in the family. On clinical examination, the patient was conscious, normocardic, normotensive, eupneic, and apyretic (36°). Abdominal examination was unremarkable. The clinical history dated back three months to the onset of chronic liquid diarrhea without mucus or blood, with three to four stools per day, diurnal, and without associated digestive manifestations, notably no abdominal pain, no externalized upper or lower digestive hemorrhage, no jaundice, no abdominal distension, and no other extradigestive manifestations. All of this evolved in a context of apyrexia and altered general condition with asthenia and weight loss. In view of this clinical picture, a biological workup was carried out, which revealed a hemoglobin level of 12.6 g/dl, a leukocyte count of 8130, and a C-reactive protein of 4. Her renal function was normal, and stool analysis revealed no ova or cysts (Table 1).

**Table 1:** Laboratory findings in our case HIV: Human immunodeficiency virus; HBsAg: Hepatitis B surface antigen; Anti-HCV: Anti-hepatitis C virus antibodies.

Parameters	Patient value	Reference range
White blood cell count (/μL)	8130	4000-11000
Eosinophilic count (/μL)	140	20-630
Lymphocytic count (/μL)	1770	1000-4800
Neutrophilic count (/μL)	5080	1400-7700
Hemoglobin (g/dL)	12.6	13-18
Creatinine (μmol/L)	55	50-90
Natremia (mmol/L)	143	136-145
Kalemia (mmol/L)	4.40	3.5-4.6
C-reactive protein (mg/L)	4	<5
Albumin (g/L)	37	35-50
Viral markers (HIV, HBsAg, and anti-HCV)	Non-reactive	
Stool for ova and cysts	Negative	

**Figure 1:**
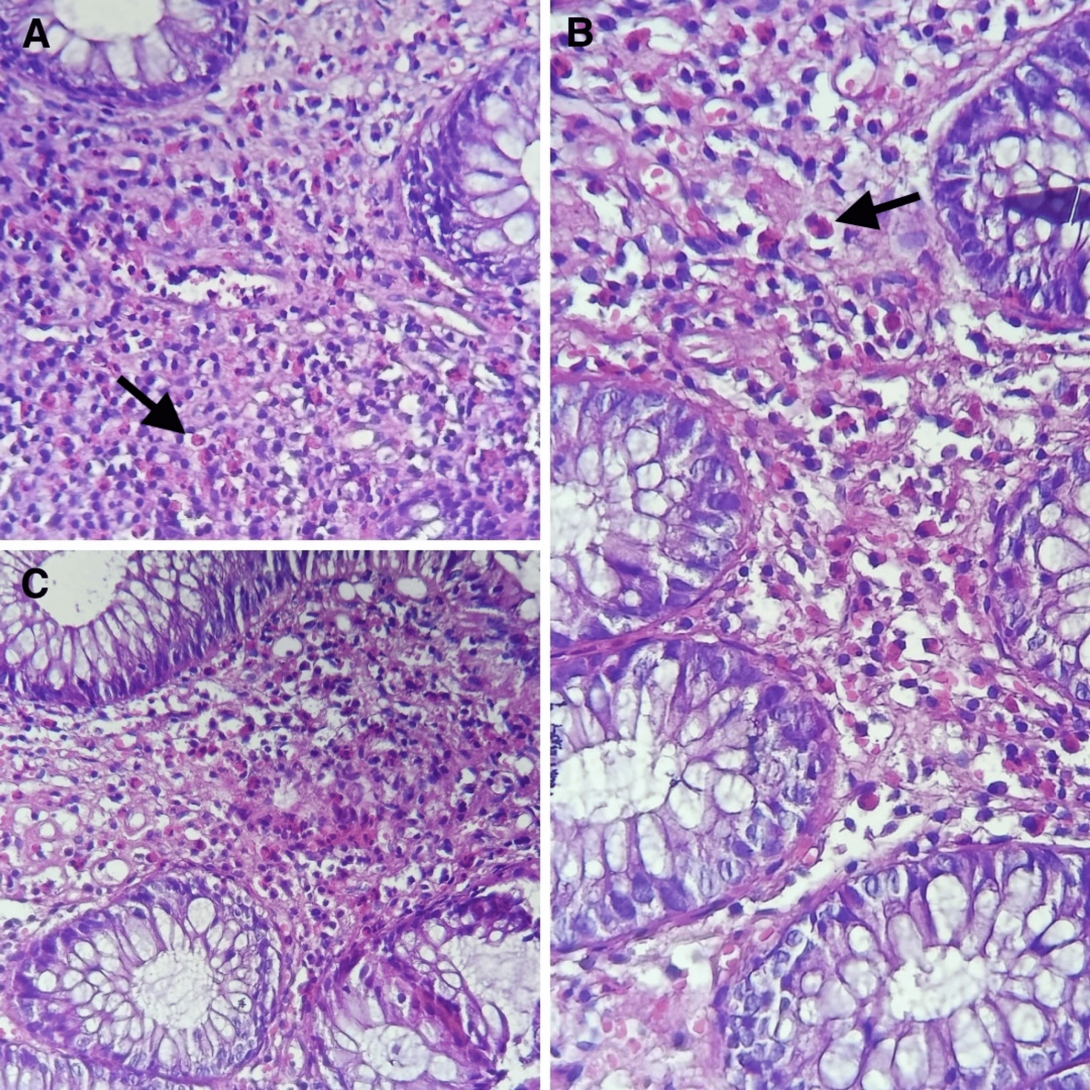
(A,B,C) Hematoxylin-eosin (H&E)-stained histological sections of the colonic mucosa showing marked eosinophil infiltration without architectural alteration, consistent with eosinophilic colitis

Endoscopy (esogastroduodenal fibroscopy and ileocolonoscopy) revealed macroscopically normal mucosa in the stomach, duodenum, terminal ileum, and colon. Anatomopathological study of the biopsies revealed subacute edematocongestive colitis rich in eosinophilic polynuclears (>100/HPF) (Figure 1).

The patient was treated with oral corticosteroids at a daily dose of 1 mg per kilogram for four weeks, followed by a gradual reduction and antihistamines (10 mg once a day) for one week. Our patient's progress was marked by a clear clinical improvement, with fewer episodes of diarrhea. The patient gradually began to regain weight.

## Discussion

EC is a rare condition usually caused by infiltration of the mucosa and can be primary or secondary [2]. In most cases, primary EC is caused by an allergic reaction, either IgE-mediated, leading to anaphylactic food allergy, or non-IgE-mediated, leading to food enteropathy. Secondary EC can result either from diseases unrelated to eosinophilic disorders, such as parasitic infections, systemic diseases, inflammatory bowel diseases, and certain medications, or from eosinophilic disorders, such as hypereosinophilic syndrome. Clinical signs are variable and nonspecific, often intermittent and interspersed with periods of remission. Diarrhea is the most common clinical sign, occurring in more than 60% of cases. Abdominal pain is also common, occurring in 60%-80% of cases, while rectal secretions occur in only 10%-20% of cases. Nausea and vomiting may also be observed in 30% of cases; minimal weight loss is possible, but a change in general health is rare [1].

Biological assessments are not of great interest due to their low sensitivity and specificity. Blood hypereosinophilia is a biological marker that, when associated with other factors, can help in the diagnosis; however, it is not always present, occurring in only 27%-75% of patients with EC, while being more frequent and more intense in cases of primary EC [3].The colonic mucosa is generally macroscopically normal in approximately 70% of cases, which is the same in our case [3].

The histological diagnosis of eosinophilic esophagitis is difficult. Colonic involvement has been little studied, unlike eosinophilic esophagitis. The lack of consensus on the diagnosis of EC has led to multiple definitions based on different criteria, varying from one author to another and from one study to another [4].

The progression of EC is poorly understood, as it has been little studied. In older children and adults, although the prognosis is good, progression to chronicity is common, with alternating flares and remissions of variable duration [5]. A few cases have been reported with bloody diarrhea or massive rectal bleeding [6]. In cases of transmural involvement of the colonic wall, complications have been published, such as perforation [7], stenosis and occlusion, intussusception [8], and volvulus [9].

It is a relatively well-known disease in children under two years of age and infants, whereas it is less studied and less common in adults, with only a few isolated cases and small series published [5]. The management criteria for EC are poorly defined, unlike for eosinophilic esophagitis, due to its poorly understood pathophysiology, rarity, possible spontaneous regression of symptoms and colitis, and lack of controlled studies. Oral corticosteroid therapy remains the cornerstone of treatment, improving symptoms and histological lesions in the vast majority of cases [10]. Budesonide is an alternative treatment that has also been shown to be effective, with prolonged remissions [11,12] and fewer side effects than conventional corticosteroid therapy, but this requires further studies for better confirmation.

## Conclusions

EC is an underdiagnosed and underestimated cause of chronic diarrhea. Diagnosis remains challenging due to the absence of standardized histological criteria and unclear physiological eosinophil thresholds in normal colonic mucosa. Unfortunately, very few studies have addressed these issues to date. Therefore, several larger case-control and cohort studies are needed to address this need.

This case highlights the importance of systematic colonic biopsies in patients with chronic diarrhea and normal endoscopic findings and demonstrates the effectiveness of oral corticosteroid therapy in achieving clinical improvement.
